# Supplementary material for: Phenolic Fingerprinting, Antioxidant, and Deterrent Potentials of Persicaria maculosa Extracts
Source: Molecules. 2020 Jul 3;25(13):3054. doi: 10.3390/molecules25133054 (PMC7411858; doi:10.3390/molecules25133054)
Supplement: Supplementary file 1 [file molecules-25-03054-s001.pdf]

Supplementary material

Article

Phenolic Fingerprinting, Antioxidant, and Deterrent Potentials of *Persicaria maculosa* Extracts

Luisa Quesada-Romero [1,2], Carlos Fernández-Galleguillos [3], Jan Bergmann [1], María-Eugenia Amorós [4], Felipe Jiménez-Aspee [5], Andrés González [4], Mario Simirgiotis [3,6,*] and Carmen Rossini [4,*]

[1] Laboratorio de Ecología Química, Instituto de Química, Pontificia Universidad Católica de Valparaíso, Avda. Universidad 330. Curauma, Valparaíso, 2340000, Chile
[2] Facultad de Ciencias para el cuidado de la Salud, Universidad San Sebastián, General Lagos 1163. Valdivia5090000, Chile.
[3] Instituto de Farmacia, Facultad de Ciencias, Universidad Austral de Chile, Valdivia 5090000, Chile,
[4] Laboratorio de Ecología Química, Facultad de Química, Universidad de la República, Gral. Flores 2124 CP 11800, Montevideo 11800, Uruguay.
[5] Departamento de Ciencias Básicas Biomédicas, Facultad de Ciencias de la Salud, Universidad de Talca, Avenida Lircay S/N, Talca 3460000, Chile.
[6] Center for Interdisciplinary Studies on the Nervous System, Universidad Austral de Chile, Campus Isla Teja 5090000, Valdivia.
* Correspondence: mario.simirgiotis@uach.cl (M.S.); crossini@fq.edu.uy (C.R.) Tel.: +056-632244369 (M.S.)

Antifeedant activity

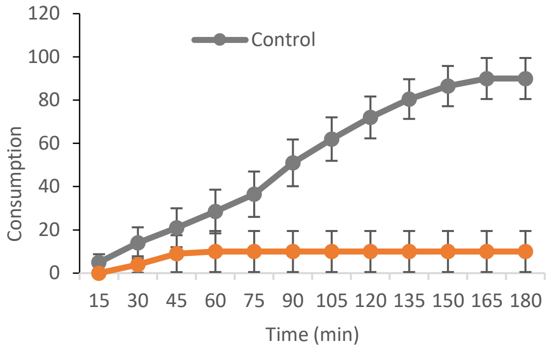

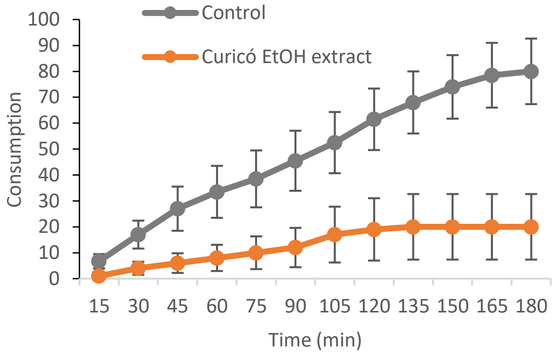

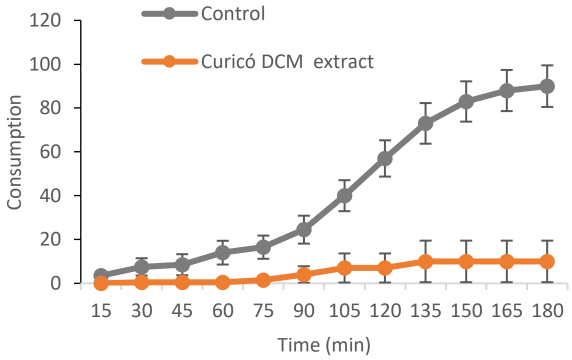

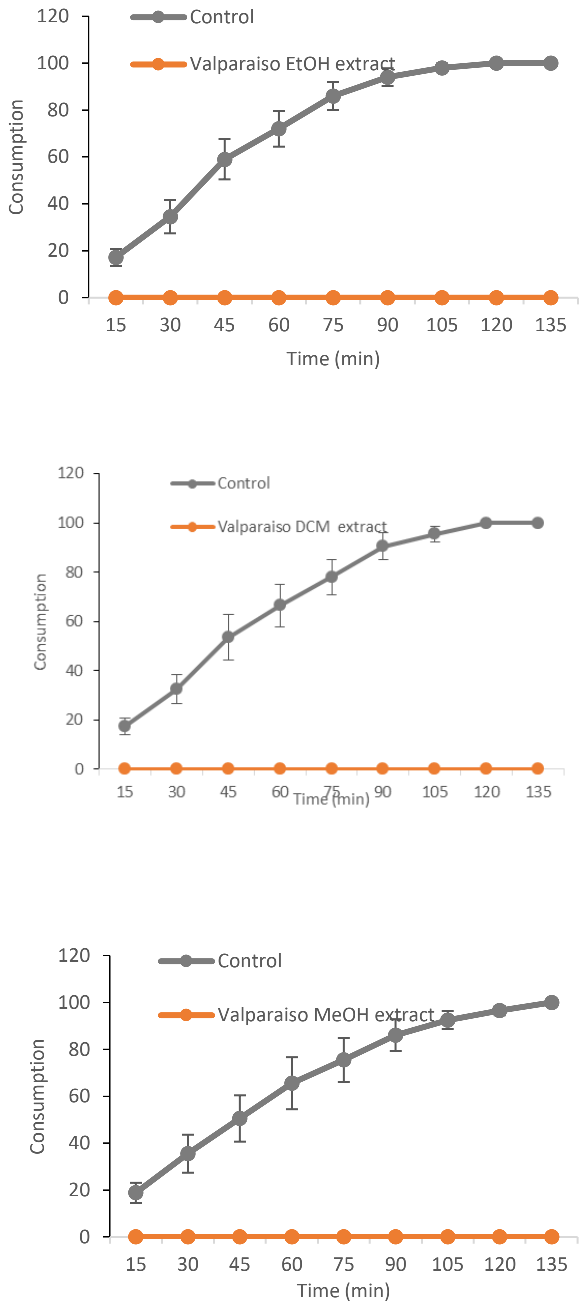

Fig S1. Leaf consumption by *Epilachna paenulata* (circles) on leaf disks treated with the corresponding solvent (Control, grey) and with the extract (orange). Consumption was different in all cases as a function of treatment, time and the interaction time*treatment (ANOVA, GLM, $p < 0.05$).

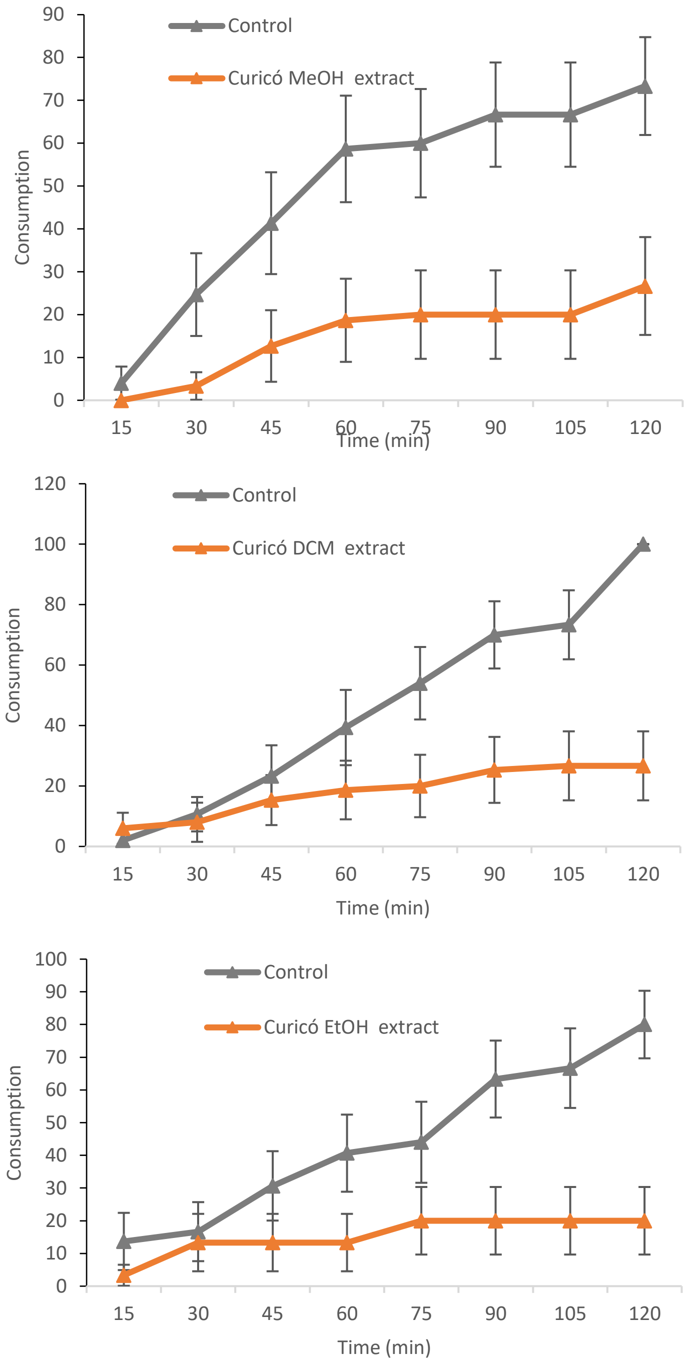

Control
Curicó MeOH extract
Consumption
Time (min)
0
10
20
30
40
50
60
70
80
90
15
30
45
60
75
90
105
120
Control
Curicó DCM extract
Consumption
Time (min)
0
20
40
60
80
100
120
15
30
45
60
75
90
105
120
Control
Curicó EtOH extract
Consumption
Time (min)
0
10
20
30
40
50
60
70
80
90
100
15
30
45
60
75
90
105
120

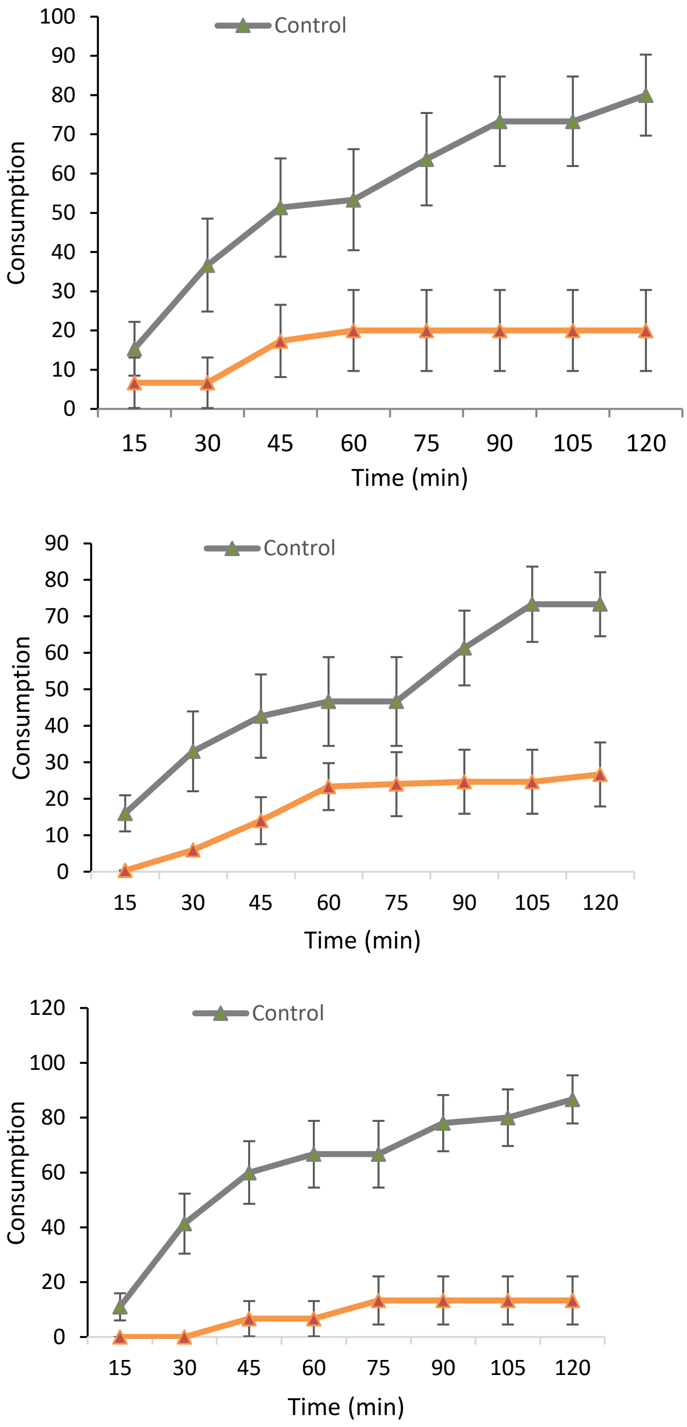

Fig S2: Leaf consumption by *Pseudaletia adultera* (triangles) on leaf disks treated with the corresponding solvent (Control, grey) and with the extract (orange). Consumption was different in all cases as a function of treatment, time and the interaction time*treatment (ANOVA, GLM, $p < 0.05$).
